# Supplementary figures and images for: Beyond gene ontology (GO): using biocuration approach to improve the gene nomenclature and functional annotation of rice S-domain kinase subfamily
Source: PeerJ. 2021 Mar 15;9:e11052. doi: 10.7717/peerj.11052 (PMC7971086; doi:10.7717/peerj.11052)

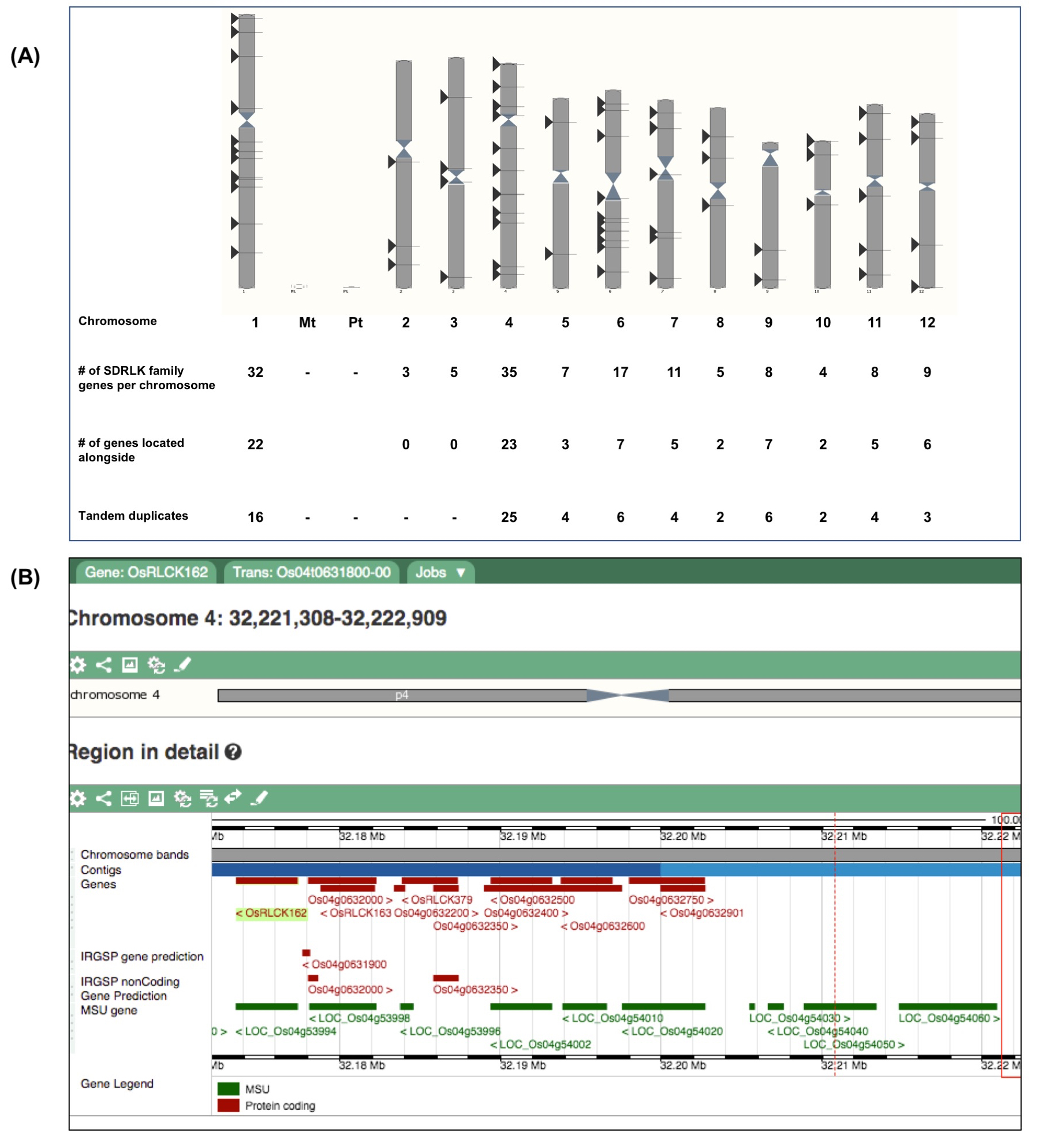

Supplement: Supplemental Information 1 — (A) Distribution of 114 SDRLK family genes on 12 rice chromosomes. (B) A close view of tandemly arranged genes on chromosome 4 showing Os04g0631800 (OsRLCK162), Os04g0632100 (OsRLCK163), Os04g0632500, Os04g0632600, and Os04g0632901 genes. [file peerj-09-11052-s001.png]

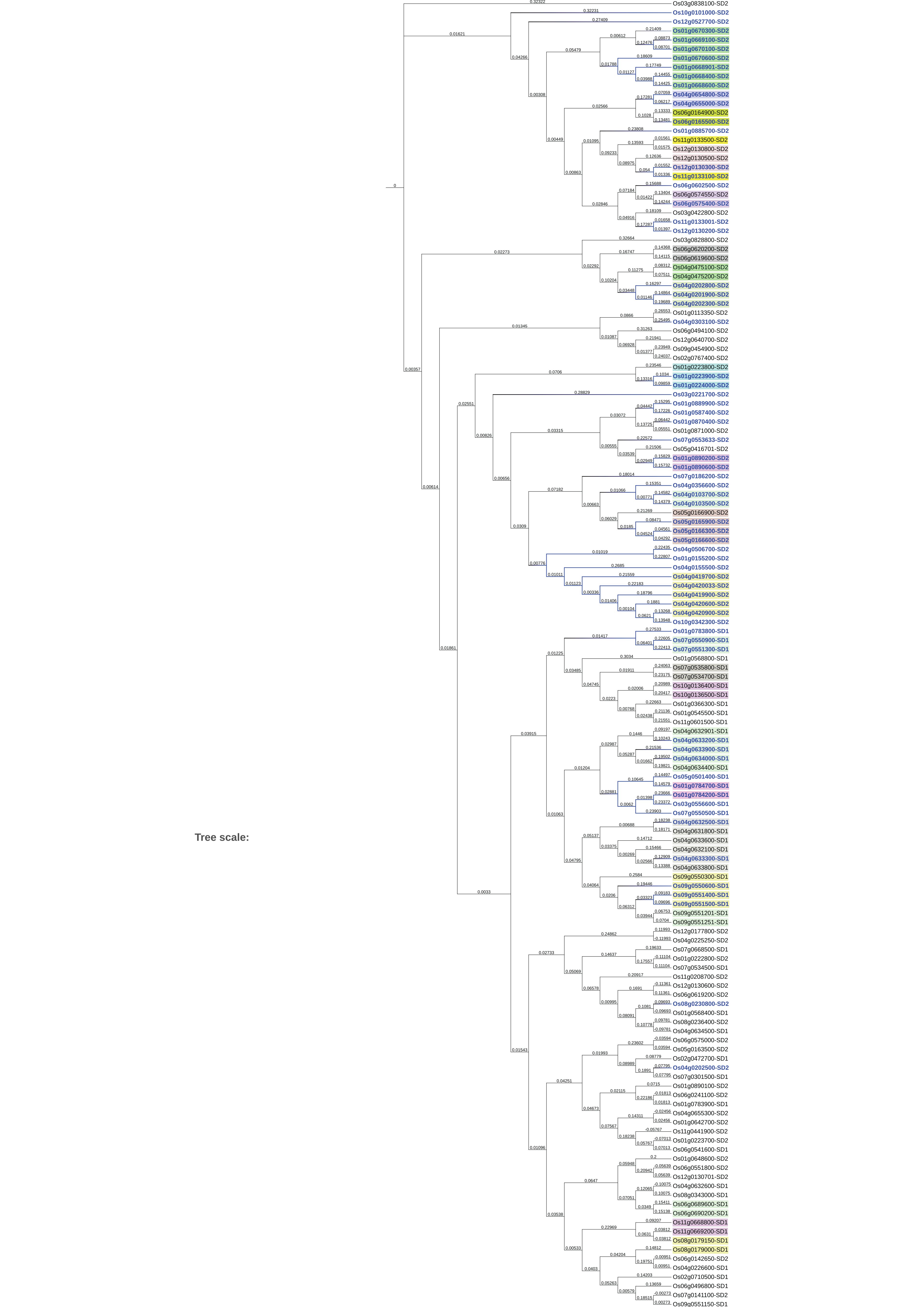

Supplement: Supplemental Information 2 — Tips were aligned to the right-hand side, and the branch length (indicative of the evolutionary distance between the sequences) information is displayed at the top of each branch. The blue color nodes and labels in the tree represent full-length RLKs. Groups of genes shaded with the same color depict tandem duplicates. [file peerj-09-11052-s002.png]

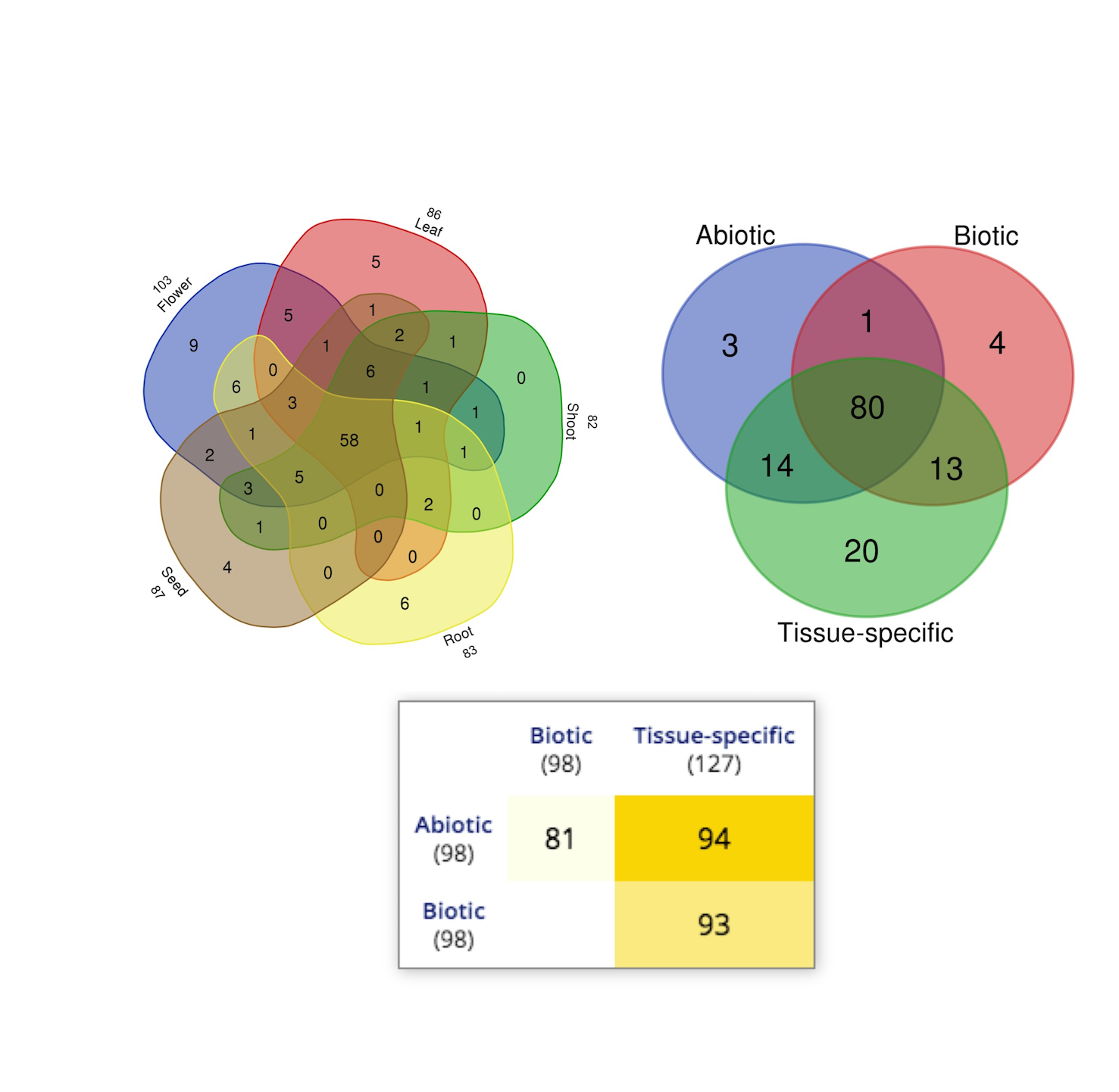

Supplement: Supplemental Information 3 [file peerj-09-11052-s003.png]
