# Supplementary material for: Beyond gene ontology (GO): using biocuration approach to improve the gene nomenclature and functional annotation of rice S-domain kinase subfamily
Source: PeerJ. 2021 Mar 15;9:e11052. doi: 10.7717/peerj.11052 (PMC7971086; doi:10.7717/peerj.11052)
Supplement: Supplemental Information 5 [file peerj-09-11052-s005.docx]

**Supplementary Table 2. A list of transcriptome datasets used in this study.**

| **Data source** | **Sample Description** | **Reference** | **# Genes** |
| --- | --- | --- | --- |
| **Baseline tissue-specific expression data** | | | |
| EMBL-EBI-Expression ATLAS  https://www.ebi.ac.uk/arrayexpress/experiments/E-GEOD-50777 | This set has data from three tissues: egg cell, pollen sperm, and microgametophyte vegetative cell of *O. sativa* L. ssp*. japonica*. | (Anderson et al. 2013) | 73 |
| EMBL-EBI-Expression ATLAS  https://www.ebi.ac.uk/arrayexpress/experiments/E-MTAB-4123 | This set has data from shoot, anther, carpel, and seed of *O. sativa* L. ssp*. japonica*. | (Zhang et al. 2014) | 43 |
| EMBL-EBI-Expression ATLAS  https://www.ebi.ac.uk/arrayexpress/experiments/E-MTAB-2037 | This data are from callus, leaf, root, seed, shoot, panicle before flowering, and panicle after flowering of *O. sativa* L. ssp*. japonica*; samples were taken between 7 days pre-flowering to 7 days post- germination. | (Sakai et al. 2011) | 102 |
| EMBL-EBI-Expression ATLAS  https://www.ebi.ac.uk/arrayexpress/experiments/E-MTAB-2039 | This data is from leaf (20 days after sowing) early inflorescence (50 days after sowing); emerging inflorescence (60 days after sowing); anther and pistil (65 days after sowing); seed 5 and 10 days after pollination; plant embryo and endosperm (85 days after sowing). | (Davidson et al. 2012) | 84 |
| **Differential gene expression data under biotic stress** | | | |
| EMBL-EBI-Expression Atlas [https://www.ebi.ac.uk/arrayexpress/experiments/E-GEOD-61833](https://www.ebi.ac.uk/arrayexpress/experiments/E-GEOD-61833/) | This data is from of *O. sativa* L. ssp*. japonica*. leaves treated with three*)* strains of *Xanthomonas oryzae pv. oryzae (Xoo*): wildtype, LPS^+^EPS^-^, and LPS^-^EPS^-^. | (Girija et al. 2017) | 18 |
| EMBL-EBI-Expression ATLAS https://www.ebi.ac.uk/arrayexpress/experiments/E-GEOD-36272 | This data is from 14 days old leaves of *O. sativa ssp. indica* cultivar IR24 and *O. sativa ssp* japonica cultivar Nipponbare. Each cultivar was inoculated with various *Xoo* strains (three wild-type, two mutants, one reduced virulence, and one non-pathogenic) and with a wildtype *Xanth*o*monas oryzae pv. oryzicola (Xoc)* strain. | N/A | 41 |
| Data extracted from http://dx.doi.org/10.1186/1471-2164-14-93 | These microarray expression data are from *O. sativa ssp. indica* cultivars IR24 (susceptible) and IRBB21(resistant) treated with *Xoo* strain PX071. | (Narsai et al. 2013) | 18 |
| EMBL-EBI-Expression ATLAS https://www.ebi.ac.uk/arrayexpress/experiments/E-MTAB-4406 | RNA-seq data are from two rice breeding lines, CL161 (resistant) and CL151 (susceptible) that were inoculated with *Burkholderia glumae,* the causal agent of bacterial panicle blight disease in rice. | (Magbanua et al. 2014) | 72 |
| EMBL-EBI-Expression ATLAS https://www.ebi.ac.uk/arrayexpress/experiments/E-MTAB-5025 | This RNA-seq dataset contains differential gene expression data from rice leaves mechanically inoculated with insect-derived Rice Stripe Virus (RSV), rice leaves mechanically inoculated with plant-derived RSV, and rice leaves fed on by viruliferous small brown plant hoppers (*Laodelphax striatellus*), the natural viral vector. | (Zhao et al. 2016). | 41 |
| ArrayExpress  https://www.ebi.ac.uk/ena/data/view/ERS2106879-ERS2106902  or E-MTAB-6402 | This RNA-seq data are from the sheath blight resistant rice variety MCR010277 (MCR) and the susceptible rice variety Cocodrie (CCDR) infected with fungal pathogen *Rhizoctonia* *solani* strain LR172 (at day 3 and Day 5 post infection). Both CCDR and MCR are long-grain, high-yielding breeding line developed in the U.S (<https://www.lsuagcenter.com)> | (Al-Bader et al. 2019) | 39 |
| **Differential gene expression data under abiotic stress** | | | |
| Chilling (cold) stress | | | |
| ArrayExpress  https://www.ebi.ac.uk/arrayexpress/experiments/E-MTAB-5941 | This RNA-seq data are from a susceptible Thaibonnet and resistant Volano cultivars of rice subjected to 0, 2, and 10 hours chilling treatment at10 °C. | (Buti et al. 2018) | 55 |
| ArrayExpress  https://www.ebi.ac.uk/arrayexpress/experiments/E-GEOD-38023 | This microarray data are from tolerant LTH and sensitive IR29 rice genotypes subjected to chilling stress for 2, 8, 24, and 48 hours as well as 24 hours of recovery. | (Zhang et al. 2012). | 59 |
| Drought stress | | | |
| ArrayExpress  https://www.ebi.ac.uk/arrayexpress/experiments/E-GEOD-41647 | This microarray expression data are from seedlings of susceptible IR20 and tolerant Dagad deshi rice genotypes subjected to drought stress for 3 and 6 hours. | unpublished | 26 |
| ArrayExpress  https://www.ebi.ac.uk/arrayexpress/experiments/E-MTAB-4994 | This dataset is from flag leaf taken at reproductive/panicle initiation stage of Nagina 22 rice variety exposed to drought stress and compared to control. | unpublished | 20 |
| ArrayExpress  https://www.ebi.ac.uk/arrayexpress/experiments/E-MTAB-7317 | This RNA-seq dataset contains differential gene expression data from Nagina 22 (drought tolerant) vs. Nonabokra (salt tolerant) rice varieties subjected to drought treatment. The samples from both varieties were collected to study early (day-0 vs day-2) response and late (day-2 vs day-3) response to drought treatment. | unpublished | 44 |
| EMBL-EBI-Expression ATLAS  https://www.ebi.ac.uk/gxa/experiments/E-MTAB-3230 | This microarray based gene expression data are from seedlings of Nagina 22 and an enhanced water-stress tolerant (ewst1) mutant of Nagina 22 grown in hydroponic culture subjected to 25% PEG stress for one hour. | (Lima et al. 2015). |  |
| Submergence |  |  |  |
| Data extracted from the supplementary file  https://www.mdpi.com/1422-0067/21/4/1292/s1 | Transcriptome data from *O. sativa* L. japonica cultivar Taikeng 9 under submergence stress with and without polar auxin transport inhibitor 2,3,5-triiodobenzoic acid (TIBA). | (Wu & Yang 2020). | 20 |
| Data extracted from the supplementary table  http://dx.doi.org/[10.3389/fpls.2016.01125](https://dx.doi.org/10.3389%2Ffpls.2016.01125) | Transcriptome data from primed (selenium-Se and salicylic acid-SA priming) and non-primed *O. sativa* L. indica cultivar Huanghuazhan 4-days-old seedlings under submergence. | (Hussain et al. 2016). | 19 |
