# Supplementary material for: Beyond gene ontology (GO): using biocuration approach to improve the gene nomenclature and functional annotation of rice S-domain kinase subfamily
Source: PeerJ. 2021 Mar 15;9:e11052. doi: 10.7717/peerj.11052 (PMC7971086; doi:10.7717/peerj.11052)
Supplement: Supplemental Information 9 [file peerj-09-11052-s009.docx]

**Supplementary Table 6.** A summary of tissue-specific gene expression of the rice SDRLK family genes.

| **Tissue** | **# of genes** | **Gene ID** |
| --- | --- | --- |
| **Gene expressed under normal growth and development (127)** | | |
| Unique Expression (26) | | |
| Leaf | 5 | Os01g0366300; Os01g0870400; Os04g0420033; Os08g0236400; Os11g0441900 |
| Root | 6 | Os04g0506700; Os05g0166900; **Os09g0550300**(RLP); **Os09g0551251**(Kinase); Os11g0133500; Os11g0668800 (Gao & Xue 2012) |
| Flower | 9 | Os01g0783900; Os01g0784200; Os01g0890200; Os04g0633800; Os07g0301500; Os07g0534500; Os07g0553633; Os08g0179150; Os09g0550600 |
| - Microgametophyte cell |  | Os01g0784200; Os07g0301500 |
| - Pollen |  | Os01g0783900; Os04g0633800; Os07g0301500; Os09g0550600 |
| - Pistil |  | Os07g0553633 |
| Seed / shoot | 4 | Os05g0501400; Os07g0141100; Os09g0551400; Os11g0601500 |
| - Embryo |  | Os11g0601500; Os07g0141100 |
| Callus | 1 | Os01g0568400 |
| Expression in two or more tissues (44) | | |
| Flower & Leaf | 5 | Os01g0784700; Os01g0885700; Os04g0226600  **Os04g0632901**; **Os04g0633200** |
| Flower & Root | 6 | Os02g0767400; Os04g0202500; **Os04g0633900**; **Os04g0634400**; Os06g0575400; Os07g0550900 |
| Flower & Seed | 2 | Os06g0620200; Os09g0551500 |
| Leaf & Shoot | 1 | Os04g0419700 |
| Leaf & Root | 1 | Os04g0356600 |
| Leaf & Seed | 1 | Os01g0155200 |
| Seed & Shoot | 1 | Os11g0133001 |
| Shoot (nodes) and flower | 1 | Os01g0890100 (PSRK2) shows high expression in the nodes after the start-up of reproductive growth (Li et al. 2018). |
| Flower, Leaf, Root, & Shoot | 1 | Os05g0166300 |
| Flower, Leaf, Seed, & Shoot | 6 | Os02g0472700; Os03g0828800; Os04g0475200; Os06g0551800 Os04g0632100; Os10g0136400 |
| Flower, Leaf, Root, & Seed | 3 | Os01g0670600; Os04g0632600; Os06g0165500 |
| Flower, Root, Seed, & Shoot | 5 | Os01g0223800* (OsESG1), shows high expression in embryo and under abiotic stress) (Pan et al. 2020); Os01g0223900*; Os01g0222800; Os01g0648600; Os11g0669200 |
| Flower, Leaf, & Shoot | 1 | Os01g0890600 |
| Flower, Leaf, & Seed | 1 | Os06g0241100 |
| Flower, Root, & Shoot | 1 | Os12g0640700 |
| Flower, Seed, & Shoot | 3 | Os01g0642700; Os04g0202800; Os11g0133100 |
| Flower, Root, & Seed | 1 | Os09g0454900 |
| Leaf, Root, & Shoot | 2 | Os01g0669100 (OsLSK1, preferential expression in nodes) (Zou et al. 2015)  Os06g0494100 (PID2), shows high expression in rice variety Digu resistant to blast disease (Wang et al. 2015) |
| Leaf, Seed, & Shoot | 2 | Os10g0136500; Os12g0130300 |
| Ubiquitous Expression in Flower, Leaf, Root, Seed, & Shoot (total 58) | | |
| Highly expressed in all tissues | 13 | **Os01g0223700**; **Os01g0224000**; Os01g0783800 (SDS2)(Fan et al. 2018); Os01g0889900; Os03g0838100; Os04g0631800; Os04g0634500; Os04g0655300; Os05g0165900; Os06g0496800; Os06g0602500; Os07g0551300; Os08g0343000 |
| Expressed in all tissues but show high expression in one or more tissue | 45 | **Preferential expression**  Leaf: Os01g0113350; Os04g0201900; Os04g0419900; Os08g0230800  Root: Os02g0710500; **Os06g0689600**; **Os06g0690200**; Os12g0130500  Shoot: Os03g0221700  Callus: Os07g0550500  Seed: Os03g0556600; Os04g0633300; Os05g0163500  Flower: Os01g0668901; Os03g0422800; Os04g0103500; Os08g0179000; Os12g0177800   - Carpel/pistil/egg: Os01g0587400; Os01g0668400; Os04g0303100; Os06g0164900; Os06g0541600; Os06g0575000; Os12g0527700 - Pollen sperm: **Os04g0420600**; **Os04g0420900** - Tetrad-microspore cells: Os01g0545500; Os04g0632500; Os05g0166600   Leaf, sheath, and root: Os07g0186200 (SIK2)  Flower and leaf: Os04g0633600  Shoot and seed: Os07g0535800  Root and shoot: Os01g0670100; Os07g0668500  Root and seed: Os07g0534700; Os10g0101000  Root and flower: Os01g0668600; **Os04g0103700**; **Os04g0202300**  Os04g0634000; Os04g0655000; Os10g0342300; Os11g0208700;  Os12g0130200; |
| **Genes not expressed** | 9 | Os01g0568800; Os04g0225250; Os04g0475100; Os05g0416701; **Os06g0619200**; **Os06g0619600**; Os06g0574550; **Os12g0130600**; **Os12g0130701** |
| **Genes expressed in seedlings or whole plant** | 8 | Evidence from differential expression datasets: Os01g0670300; Os01g0871000: Os04g0155500; Os04g0654800; Os06g0142650; Os09g0551201; and Os12g0130800.  Os09g0551150: expressed under oxidative stress (Liu et al. 2010) |
| Total | 144 |  |
