# Supplementary material for: Beyond gene ontology (GO): using biocuration approach to improve the gene nomenclature and functional annotation of rice S-domain kinase subfamily
Source: PeerJ. 2021 Mar 15;9:e11052. doi: 10.7717/peerj.11052 (PMC7971086; doi:10.7717/peerj.11052)
Supplement: Supplemental Information 10 [file peerj-09-11052-s010.docx]

**Supplementary Table 7.** A summary of changes in the expression of the rice SDRLK family genes in response to biotic and abiotic stresses.

| **Stress treatment** | **Differential expression of SDRLK family genes** |
| --- | --- |
| **Abiotic stress response genes** | |
| Oxidative stress | Os09g0551150: up-regulated |
| Drought | Up-regulated: Os01g0366300; **Os04g0632100**; **Os04g0633600**;  Os12g0130800  Down-regulated: Os01g0223700; Os04g0420033; **Os04g0634400**; **Os04g0634500**; Os06g0165500 |
| Submergence | Up-regulated: Os01g0587400; Os01g0670600; Os02g0767400; Os05g0166900; Os05g0501400; Os06g0575000; Os07g0141100; Os12g0130500  Down-regulated: Os06g0496800; Os04g0202300; **Os04g0419700; Os04g0420600** |
| Chilling | Up-regulated: Os12g0640700  Down-regulated: Os04g0633300; Os06g0142650; Os06g0541600; |
| Drought and submergence | Os01g0670100: up-regulated in response to drought/submergence  Os06g0241100: up-regulated in response to drought; up-regulated in response to submergence in Indica cultivar Huanghuazhan, but down-regulated in japonica cultivar Taikeng 9.  Os06g0575400: up-regulated in response to submergence; down-regulated in response to drought.  Os08g0179000 down-regulated in response to drought/submergence. |
| Submergence and chilling | Os06g0164900 up-regulated in response to submergence/chilling |
| Drought and chilling | Os01g0885700 up-regulated in response to both drought/chilling.  Os03g0422800 down-regulated in response to drought; up-regulated in response to both short- and long-term chilling stress |
| Drought, submergence, and chilling (and other stresses) | Os01g0223800 (*ESG1*): its expression is induced by abiotic stress and treatment with PEG, NaCl and ABA. up-regulated in response to drought and shown to provide drought tolerance (Pan et al. 2020).  Os06g0690200: up-regulated in response to drought/chilling; down-regulated under submergence.  Os07g0186200 (*SIK2*): its expression is induced by NaCl, drought, cold, dark, and abscisic acid treatment. Its overexpression results in drought and salt tolerance and delays dark-Induced leaf senescence (Chen et al. 2013).  Os07g0535800: up-regulated in response to drought/chilling/submergence |
| **Biotic stress response genes** | |
| *Xanthomonas oryzae pv. oryzae (Xoo)*  (bacterial blight disease) | Up-regulated: Os03g0838100; Os04g0226600 |
| *B. glumae*  (panicle blight disease) | Up-regulated: Os01g0113350; Os01g0668600; Os04g0633200;  Os09g0551400; Os11g0133001; Os11g0441900  Down-regulated: Os04g0475200 |
| *R. solani*  (sheath blight disease) | Up-regulated: Os02g0472700  Down-regulated: Os01g0668400; Os07g0550500 |
| Rice Stripe Virus (RSV)  (stripe disease) | Up-regulated: Os04g0103500  Down-regulated: Os11g0133100 |
| *Xoo* and *R. Solani* | Down-regulated: Os01g0784700 |
| *B. glumae* and RSV | Up-regulated: Os12g0130200 |
| *M. grisea*  (blast disease). | Up-regulated: Os06g0494100 (*PID2*) is known to confer a race‐specific resistance to *M. grisea (Wang* et *al. 2015)* |
| **Common genes in abiotic and biotic stress response** | |
| Gene ID | Differential gene expression under stress conditions |
| Os01g0155200 | Up-regulated in response to drought/chilling/*B. glumae* |
| Os01g0222800 | Up-regulated in response to drought/*B. glumae* |
| Os01g0223900 | Down-regulated in response to chilling; up-regulated: in response to *B. glumae* |
| Os01g0224000 | Up-regulated in response to drought/chilling/Rice Stripe Virus |
| Os01g0668901 | Up-regulated in response to Xoo/*R. solani;* down-regulated under submergence |
| Os01g0669100 (LSK1) | Up-regulated: in response to chilling/submergence/*Xoo*  Down-regulated in response to drought  Over-expression of a truncated version of *OsLSK1* (including the extracellular and transmembrane domain) resulted in 55.8% increase in grain yield (due to increase in primary branches per panicle as well as grains per primary branch) (Zou et al. 2015). |
| Os01g0783800 (SDS2) | Up-regulated under submergence; and in rice transgenic lines over-expressing SUB1A, ERF67 and ERF66 transcription factors (Lin et al. 2019).  -It is shown to controls programmed cell death and provides resistance to *Magnaporthe oryzae(*Fan *et al. 2018)*. |
| Os01g0889900 | Up-regulated in response to *B. glumae*/*R. solani*/submergence  Down-regulated in response to chilling |
| Os01g0890600 | Down-regulated in response to chilling/Rice Stripe Virus |
| Os02g0710500 | Down-regulated in response to drought  Up-regulated in response to submergence/ Rice Stripe Virus/*R. solani* |
| Os03g0221700 | Up-regulated in response to submergence/*B. glumae* |
| Os03g0556600 | Up-regulated: in response to long-term chilling stress/ Rice Stripe Virus  Down-regulated: in response to *B. glumae*. |
| Os04g0103700 | Up-regulated: in response to drought/submergence/*Xoo.* |
| Os04g0201900 | Up-regulated: in response to drought/ *B. glumae*  Down-regulated: in response to *R. solani.* |
| Os04g0419900 | Up-regulated: in response to chilling/ submergence/*Xoo*  Down-regulated: in response to drought |
| Os04g0420900 | Up-regulated: in response to drought/chilling/Rice Stripe Virus |
| Os04g0631800 | Up-regulated: in response to drought/*Xoo*  Down-regulated: in response to *R. solani* |
| Os04g0632600 | Up-regulated: in response to drought/chilling/*Xoo* |
| Os04g0632901 | Down-regulated: in response to *R. solani*/ drought during early phase  up-regulated in response to prolong drought |
| Os04g0655300 | Down-regulated: in response to drought/submergence  Up-regulated: in response to *Xoo* |
| Os05g0166300 | Down-regulated: in response to drought  Up-regulated under submergence/short-term chilling/*Xoo* |
| Os06g0602500 | Up-regulated in response to drought/chilling/*Xoo*  Down-regulated under submergence |
| Os06g0689600 | Down-regulated under submergence  Up-regulated in response to drought/*Xoo/B. glumae* |
| Os07g0534500 | Down-regulated: in response to drought  Up-regulated in response to chilling/*Xoo* |
| Os07g0534700 | Down-regulated under submergence/ *B. glumae*  Up-regulated in response to drought/chilling/*R. solani* |
| Os07g0550900 | Up-regulated in response to drought/ Rice Stripe Virus/*B. glumae* |
| Os07g0551300 | Up-regulated in response to drought/submergence  Down-regulated in response to *Xoo* |
| Os08g0343000 | Down-regulated under submergence  Up-regulated in response to *Xoo* |
| Os09g0454900 | Up-regulated in response to drought/submergence/ *Xoo/*Rice Stripe Virus  It is a direct target of SUB1A and ERF66 transcription factors (Lin et al. 2019) |
| Os10g0101000 | Up-regulated in response to chilling/submergence/*Xoo* |
| **Os10g0136400**, **Os10g0136500** | Down-regulated in response to chilling stress  Up-regulated in response to *Xoo* |
| Os12g0130300 | Up-regulated in response to drought  Down-regulated in response to Rice Stripe Virus. |
| Os12g0527700 | Down-regulated in response to drought/chilling/*Xoo/* Rice Stripe Virus  Up-regulated under submergence and in the rice transgenic lines over-expressing SUB1A, ERF67 and ERF66 transcription factors. |
